# Motor imagery has a priming effect on motor execution in people with multiple sclerosis

**DOI:** 10.3389/fnhum.2023.1179789

**Published:** 2023-09-07

**Authors:** Andrea Tacchino, Ludovico Pedullà, Jessica Podda, Margherita Monti Bragadin, Mario Alberto Battaglia, Ambra Bisio, Marco Bove, Giampaolo Brichetto

**Affiliations:** ^1^Scientific Research Area, Italian Multiple Sclerosis Foundation, Genoa, Italy; ^2^Department of Physiopathology, Experimental Medicine, and Public Health, University of Siena, Siena, Italy; ^3^Section of Human Physiology, Department of Experimental Medicine, University of Genoa, Genoa, Italy; ^4^Centro Polifunzionale di Scienze Motorie, University of Genoa, Genoa, Italy; ^5^IRCCS Policlinico San Martino, Genoa, Italy; ^6^AISM Rehabilitation Service, Italian Multiple Sclerosis Society, Genoa, Italy

**Keywords:** motor imagery, priming, multiple sclerosis, pointing movement, anisochrony

## Abstract

Priming is a learning process that refers to behavioral changes caused by previous exposure to a similar stimulus. Motor imagery (MI), which involves the mental rehearsal of action representations in working memory without engaging in actual execution, could be a strategy for priming the motor system. This study investigates whether MI primes action execution in Multiple Sclerosis (MS). Here, 17 people with MS (PwMS) and 19 healthy subjects (HS), all right-handed and good imaginers, performed as accurately and quickly as possible, with a pencil, actual or mental pointing movements between targets of small (1.0 × 1.0 cm) or large (1.5 × 1.5 cm) size. In actual trials, they completed five pointing cycles between the left and right targets, whereas in mental trials, the first 4 cycles were imagined while the fifth was actually executed. The fifth cycle was introduced to assess the MI priming effect on actual execution. All conditions, presented randomly, were performed with both dominant (i.e., right) and non-dominant arms. Analysis of the duration of the first 4 cycles in both actual and mental trials confirmed previous findings, showing isochrony in HS with both arms and significantly faster mental than actual movements (anisochrony) in PwMS (*p* < 0.01) [time (s); HS right: actual: 4.23 ± 0.15, mental: 4.36 ± 0.16; left: actual: 4.32 ± 0.15, mental: 4.43 ± 0.18; PwMS right: actual: 5.85 ± 0.16, mental: 5.99 ± 0.21; left: actual: 6.68 ± 0.20, mental: 5.94 ± 0.23]; anisochrony in PwMS was present when the task was performed with the non-dominant arm. Of note, temporal analysis of the fifth actual cycle showed no differences between actual and mental trials for HS with both arms, whereas in PwMS the fifth actual cycle was significantly faster after the four actual cycles for the non-dominant arm (*p* < 0.05) [time (s); HS right: actual: 1.03 ± 0.04, mental: 1.03 ± 0.03; left: actual: 1.08 ± 0.04, mental: 1.05 ± 0.03; PwMS right: actual: 1.48 ± 0.04, mental: 1.48 ± 0.06; left: actual: 1.66 ± 0.05, mental: 1.48 ± 0.06]. These results seem to suggest that a few mental repetitions of an action might be sufficient to exert a priming effect on the actual execution of the same action in PwMS. This would indicate further investigation of the potential use of MI as a new motor-cognitive tool for MS neurorehabilitation.

## Introduction

Priming is a learning process that refers to behavioral changes in the identification, production, or classification of a stimulus caused by previous exposure to the same or a similar stimulus (Tulving and Schacter, 1990). Priming, which often occurs after a single learning episode, is a type of implicit learning such as skill learning, which is an incremental process that usually depends on multiple repetitions (Hauptmann and Karni, 2002).

Psychologists have long studied various types of priming (e.g., perceptual and conceptual), and priming targeting the motor cortex is an interesting research topic in the field of motor control (Stoykov et al., 2017). This interest has been fueled primarily by its potential therapeutic role in improving motor behavior (Ward and Cohen, 2004); in fact, it could be part of a restorative rehabilitation approach, a therapeutic strategy aiming to improve function by targeting underlying neural mechanisms (e.g., increased excitability and normalization of inhibition) that vary depending on the priming method (Pomeroy et al., 2011). The most relevant motor priming methods for neurorehabilitation include stimulation-based priming (Dafotakis et al., 2008; Bolognini et al., 2011; Nair et al., 2011), action observation (Fadiga et al., 1995), manipulation of sensory inputs (Muellbacher et al., 2002; Beekhuizen and Field-Fote, 2008), movement-based priming (Jeannerod, 1995; Stinear and Byblow, 2004; Stinear et al., 2008, 2014; Stoykov and Stinear, 2010), and pharmacology-based priming (Martinsson et al., 2007).

Motor imagery (MI) is another promising strategy for priming the motor system (Facchini et al., 2002; Lotze and Cohen, 2006; Simmons et al., 2008; Li et al., 2009). MI is a mental process during which a subject rehearses the representation of a given motor act in working memory without engaging in its actual execution (Decety and Grèzes, 1999; Frank and Schack, 2017). Neuroimaging studies have shown that the neural mechanisms of MI overlap substantially with the mechanisms of actual execution (Grèzes and Decety, 2000; Jeannerod, 2001; Ehrsson et al., 2003; Munzert et al., 2009; Hardwick et al., 2018).

Interestingly, MI and action execution have a direct influence on each other (Yágüez et al., 1998; Boschker et al., 2000; Sirigu and Duhamel, 2001; Allami et al., 2007; Louis et al., 2008) and mentally rehearsing specific actions could lead to improved actual execution of the same actions even after a few repetitions of MI (Persichetti et al., 2020). These findings indicate that MI is able to prime a subsequent actual action (Li et al., 2005, 2009; Ramsey et al., 2010; Anwar et al., 2011) and, in line with other recent studies (Senders et al., 2012; Braun et al., 2013; Hanson and Concialdi, 2019; Gil-Bermejo-Bernardez-Zerpa et al., 2021), support the interest of clinicians in MI as a priming method and treatment strategy for neurological diseases such as Multiple Sclerosis (MS) (Seebacher et al., 2019; Kahraman et al., 2020).

MS is a chronic disease characterized by motor and cognitive symptoms (Compston and Coles, 2008) due to demyelination and axonal damage leading to loss of neuronal synchronization and functional disconnection between brain relays. People with MS (PwMS) show anisochrony (i.e., temporal uncoupling) between actual and mental movements of both upper and lower limbs (Tacchino et al., 2013, 2018; Podda et al., 2020) and dependence on fatigue, cognitive deficits, mood disorders, and disease severity (Heremans et al., 2012; Tabrizi et al., 2014; Tacchino et al., 2018). Although promising findings seem to indicate that MI improves walking, balance, fatigue, mood, and quality of life in MS (Seebacher et al., 2018, 2019; Kahraman et al., 2020), there is still limited knowledge on how MI primes actual movements in PwMS.

The present study aims to investigate whether MI primes action execution in MS. As movement speed is one of the main targets of treatment in MS (Steens et al., 2012), here we explore whether MI is able to speed up actual movements in PwMS (Avanzino et al., 2009). Adopting a modified version of the pointing task used in Tacchino et al. (2013), we ascertain the dependence on both dominant and non-dominant arms. Specifically, we expect that if MI is performed faster than the actual execution (Tacchino et al., 2013), the actual movements executed after MI will be faster in PwMS. This would shed new light on the potential role of MI practice as a skill-learning method for rehabilitative interventions in MS (Hauptmann and Karni, 2002; Seebacher et al., 2023).

## Materials and methods

### Participants

The sample size was determined from the study by Tacchino et al. (2013) using the values of the Index of Performance (IP) (a dimensionless measure of the participants’ mental movement ability) of the left arm (PwMS: N = 14, mean IP = 1.245, standard deviation = 0.280; healthy subjects, HS: N = 19, mean IP = 0.975, standard deviation = 0.235). Assuming 80% power and a 5% (two-sided) level of significance, the planned sample size was at least 14 PwMS and 18 HS. Here, 17 PwMS and 19 HS took part in the study. PwMS were recruited among the outpatients of the Genoa Rehabilitation Service of the Italian Multiple Sclerosis Society (AISM). Inclusion criteria for PwMS were clinically defined MS according to the McDonald criteria (Thompson et al., 2018), a disease-stable phase without relapses in the last 3 months, all disease courses, and an Expanded Disability Status Scale (EDSS) ≤ 6.5 (Kurtzke, 1983).

Subjects were included if they were right-handed as determined by the Edinburgh Handedness Inventory (Oldfield, 1971) and had no upper limb impairment or cognitive disorders as evaluated through an Ashworth scale score < 1 in both arms (Bohannon and Smith, 1987) or a Mini-Mental State Examination (MMSE) score < 24 (Folstein et al., 1975), respectively. Subjects with a history of severe psychiatric disorders as indicated by the Diagnostic and Statistical Manual of Mental Disorders, Fifth Edition (DSM-5) criteria (American Psychiatric Association, 2013), blurred vision, or cardiovascular and respiratory disorders were excluded. The Kinaesthetic and Visual Imagery Questionnaire (KVIQ) was administered to assess the vividness of MI (Malouin et al., 2007). PwMS were also assessed with the Modified Fatigue Impact Scale (MFIS) (Flachenecker et al., 2002) to gather information on the level of fatigue perception.

All subjects provided written, informed consent. The local ethics committee approved the study. All procedures were carried out in accordance with relevant guidelines and regulations (World Medical Association, 2013).

### Experimental protocol

We adopted a modified version of the task previously proposed by Tacchino et al. (2013). The experiment took place in a sound-attenuated room. The participants were seated comfortably in an adjustable chair in front of a table on which an A4-sized sheet of paper was placed at a distance of 20 cm from the participant’s chest. As previously described in Tacchino et al. (2013), three identical black square targets formed a hypothetical equilateral triangle with a side length of 20 cm. The vertex (start target, ST) was positioned toward the subject, while the base was located on the opposite side (left target, LT; right target, RT). On each trial, the target size could be small (1.0 × 1.0 cm) or large (1.5 × 1.5 cm).

**Figure 1:**
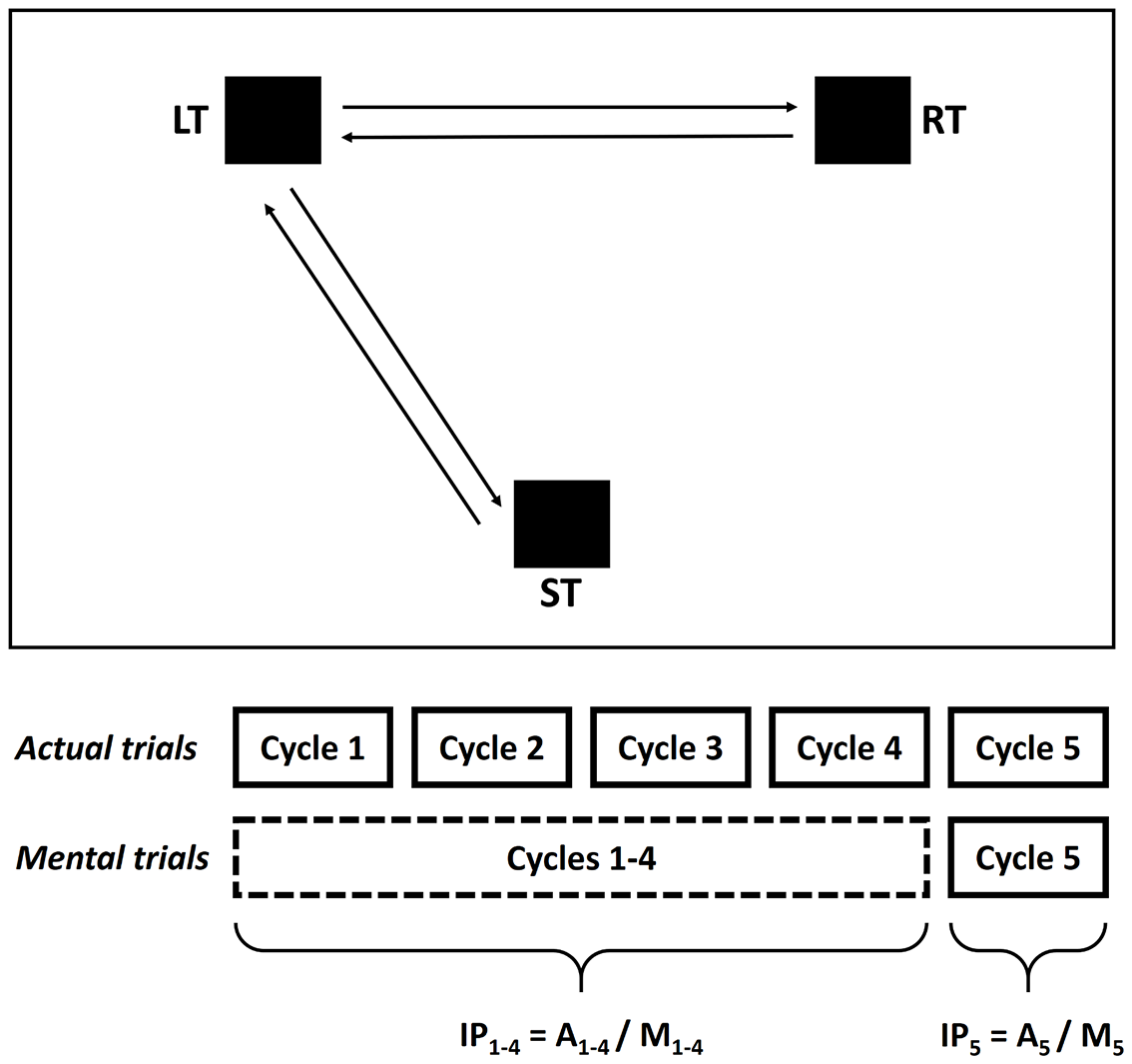
Mental and actual tasks of the experimental protocol. Subjects were asked to actually point or to imagine pointing between the three targets (ST, LT, and RT) while holding a pencil with their dominant or non-dominant arm. The targets were placed at the vertices of a hypothetical equilateral triangle (side: 20 cm). Before beginning the actual and mental trials, the subjects positioned the tip of the pencil in the center of the ST and waited for the go signal. The actual trials consisted of three phases: (1) start of the actual movement (ST-LT), (2) five actual cycles LT-RT-LT, and (3) return of actual movement (LT-ST); the mental trials consisted of four phases: (1) start of the actual movement (ST-LT), (2) four mental cycles LT-RT-LT, (3) one actual cycle LT-RT-LT, and (4) return of actual movement (LT-ST). Two indices of performance were calculated based on the first four cycles (IP_1-4_ = A_1-4_/M_1-4_) and the fifth cycle (IP_5_ = A_5_/M_5_) respectively.

The participants were asked to perform actual or imagined movements of pointing between targets with a pencil as accurately and quickly as possible (Maruff et al., 1999; Tacchino et al., 2013). Each trial started with the tip of the pencil positioned at the center of the ST. In all trials, at the “go” signal, the subjects performed an actual pointing movement toward LT; then, before returning to the ST, they completed actual (A) or mental (M) cycles of pointing movements between LT and RT (each cycle: LT-RT-LT), depending on the trial type. Specifically (Figure 1; Supplementary materials S1, S2):

- *actual trials* consisted of three phases: (1) initiation of actual movement (ST-LT), (2) five actual cycles (LT-RT-LT) and (3) return of actual movement (LT-ST).
- *mental trials* consisted of four phases: (1) initiation of actual movement (ST-LT), (2) four mental cycles (LT-RT-LT), (3) one actual cycle (LT-RT-LT), and (4) return of actual movement (LT-ST).

For the mental trials, we asked participants to feel themselves performing the task from a first-person perspective (kinesthetic internal imagery) rather than imagining that they were watching themselves do it (visual external imagery), as this has been demonstrated to be necessary for motor system engagement (Stinear et al., 2006).

The fifth cycle, which was actually executed in both actual and mental trials, was introduced to evaluate whether MI exerts a priming effect on actual execution.

Before the experiment, the participants were given complete information about the experimental procedures. Then, they familiarized themselves with all conditions: *arm* (right, left), *type* (actual, mental), and *target size* (small, large). The aim of the study was explained at the end of the experiment in order to prevent bias effects.

After 15 min of rest, the experiment started, and participants performed one trial for each condition (e.g., *arm*: right; *type*: mental; *target size*: small) for a total of eight trials, with 45 s of rest between two consecutive trials. All conditions were presented randomly to the subjects.

No feedback regarding their performance was given to the participants during the familiarization or experimental trials.

### Data analysis

The duration of pointing movements was calculated from the trajectory of a retroreflective spherical marker placed on the tip of the pencil. The kinematics of the marker were recorded by a system of six optoelectronic cameras (SMART, BTS Bioengineering; Milan, Italy, 100 Hz). Pointing movements were considered precise if the pen hit the target.

For the actual trials, the duration of each cycle was calculated based on the trajectory of the retroreflective spherical marker. However, for the mental trials, only the total duration of the first four mental cycles and the duration of the fifth actual cycle were calculated, because it was not possible to record mental time cycle by cycle.

In addition, as in Tacchino et al. (2013), an index of performance (IP) measuring isochrony (i.e., temporal equivalence) between actual and mental pointing movements was calculated as the ratio between the durations of the first 4 cycles in actual trials (A_1–4_) and the four mental cycles in mental trials (M_1–4_) (IP_1-4_ = A_1–4_/M_1–4_) (Table 1; Figure 1).

**Table 1:** Indices of performance.

**IP**_**1–4**_ **= A**_**1–4**_**/M**_**1–4**_	
IP_1–4_ ~ 1	*Isochrony*: similar duration (i.e., correct estimation) between mental and actual trials
IP_1–4_ < 1	*Anisochrony*: longer (i.e., overestimation) mental than actual trials
IP_1–4_ > 1	*Anisochrony*: shorter (i.e., underestimation) mental than actual trials

**IP5 = A5/M5**	
IP_5_ ~ 1	*Isochrony*: similar durations between the fifth cycle of mental and actual trials
IP_5_ < 1	*Slowdown*: longer fifth cycle in mental than actual trials
IP_5_ > 1	*Speeding up*: shorter fifth cycle in mental than actual trials

The table reports the definitions of the two indices of performance (IP_1-4_ = A_1-4_/M_1-4_, IP_5_ = A_5_/M_5_) and the meaning of the associated values (i.e., IP_1-4_ =, < or > 1 and IP_5_ =, < or > 1).

A second index of performance, measuring whether MI sped up actual movements, was calculated as the ratio between the durations of the fifth cycle in actual trials (A_5_) and the fifth cycle in mental trials (M_5_) (IP_5_ = A_5_/M_5_) (Table 1; Figure 1).

### Statistical analysis

All the variables considered were normally distributed (*Shapiro–Wilk W test*), and their variance was equivalent (*Levene’s test*). The statistical analysis consisted of three steps.

In the first step, we performed a repeated measure analysis of variance (RM-ANOVA) on the 5 cycles of the actual trials in order to evaluate their temporal consistency and exclude fatigue or learning effects. A RM-ANOVA was carried out for *group* (PwMS, HS), *arm* (right, left), and *target size* (small, large) conditions. A similar analysis could not be performed for imagined movements.

Second, we tested whether the total duration of the first 4 cycles in both actual and mental trials was modulated as a function of the arm, type (actual, mental), and target size; an ANOVA with *group* as a between-subjects factor and *arm*, *type*, and *target size* as within-subjects factors was performed. To better examine participants’ mental movement ability, an ANOVA with *group* as a between-subjects factor and *arm* and *target size* as within-subjects factors was performed on IP_1-4_.

Third, we tested whether the duration of the fifth cycle in both actual and mental trials was modulated as a function of arm, type, and target size; an ANOVA with *group* as a between-subjects factor and *arm, type,* and *target size* as within-subjects factors was performed. To better examine the potential MI priming effect on actual execution, an ANOVA with *group* as a between-subjects factor and *arm* and *target size* as within-subjects factors was performed on IP_5_.

*Post hoc* differences were assessed using the Newman–Keuls test, and significance was accepted at *p* < 0.05.

Statistical and descriptive analyses (mean, standard deviation, and standard errors) were run using STATISTICA 7.1.

## Results

### Participants

The PwMS group (six men and 11 women; 13 with a relapsing–remitting disease course and four with a secondary progressive disease course) had a mean age of 51.06 ± 12.24 years, a mean EDSS of 4.06 ± 1.50, and a mean disease duration of 10.76 ± 7.14 years. The HS group (11 men and eight women) had a mean age of 47.05 ± 7.31 years. No significant differences were found between the groups for age (*t* = 1.21, *p* = 0.24) and KVIQ (PwMS: 129.65 ± 23.23; HS: 133.63 ± 20.87; *t* = 7.12; *p* = 0.09); the KVIQ indicated that the participants were good imaginers. All the subjects were right-handed and had an Ashworth scale score of less than 1 in both arms and an MMSE score > 26. Moreover, PwMS reported a low level of fatigue (MFIS = 29.24 ± 12.37).

### Spatial precision and temporal consistency in actual trials

The analysis of spatial accuracy and temporal consistency within the cycles composing a trial was possible only for actual movements. PwMS and HS met the task requirements concerning spatial precision, as they missed an insignificant number of targets (<0.3%; the total number of actual movements = 1,440; i.e., 36 participants × 2 target sizes × 2 arms × 10 movements within each trial).

RM-ANOVA revealed temporal consistency through the five cycles of the actual trials for group, arm, and target size. As expected (Tacchino et al., 2013), only main effects were found for group [PwMS: 1.58 ± 0.02 s; HS: 1.07 ± 0.01 s; *F* (1,136) = 154.90, *p* < 0.001], arm [right: 1.26 ± 0.02 s; left: 1.36 ± 0.02 s; F (1,136) = 6.33, *p* < 0.05] and target size [small: 1.35 ± 0.02 s; large: 1.25 ± 0.02 s; F (1,136) = 5.66, *p* < 0.05]; no significant interactions were found.

### Temporal characteristics of actual and mental movements

A statistical analysis of the total duration of the first 4 cycles in both actual and mental trials confirmed previous findings (Tacchino et al., 2013). ANOVA revealed the main effects of the group, showing that PwMS were slower than HS [PwMS: 6.14 ± 0.11 s; HS: 4.34 ± 0.08 s; F (1,136) = 125.88, *p* < 0.001].

Moreover, we found a significant interaction between group and type [F (1,136) = 7.61, *p* < 0.01]. *Post hoc* analysis revealed a significant difference between PwMS and HS for both actual and mental durations (for both, *p* < 0.001); in addition, actual movements of PwMS were significantly longer than mental movements (PwMS actual: 6.32 ± 0.14 s, mental: 5.96 ± 0.15 s; HS actual: 4.28 ± 0.10 s, mental: 4.40 ± 0.12 s; *p* < 0.01).

There was also a significant interaction between type and arm [F (1,136) = 5.14, *p* < 0.05]. *Post hoc* analysis revealed a significant difference between actual and mental durations for the left arm, showing a temporal discrepancy between actual and mental movements only in the non-dominant arm (actual right: 5.05 ± 0.16 s, left: 5.44 ± 0.18 s; mental right: 5.13 ± 0.16 s, left: 5.14 ± 0.16 s; *p* < 0.05).

**Figure 2:**
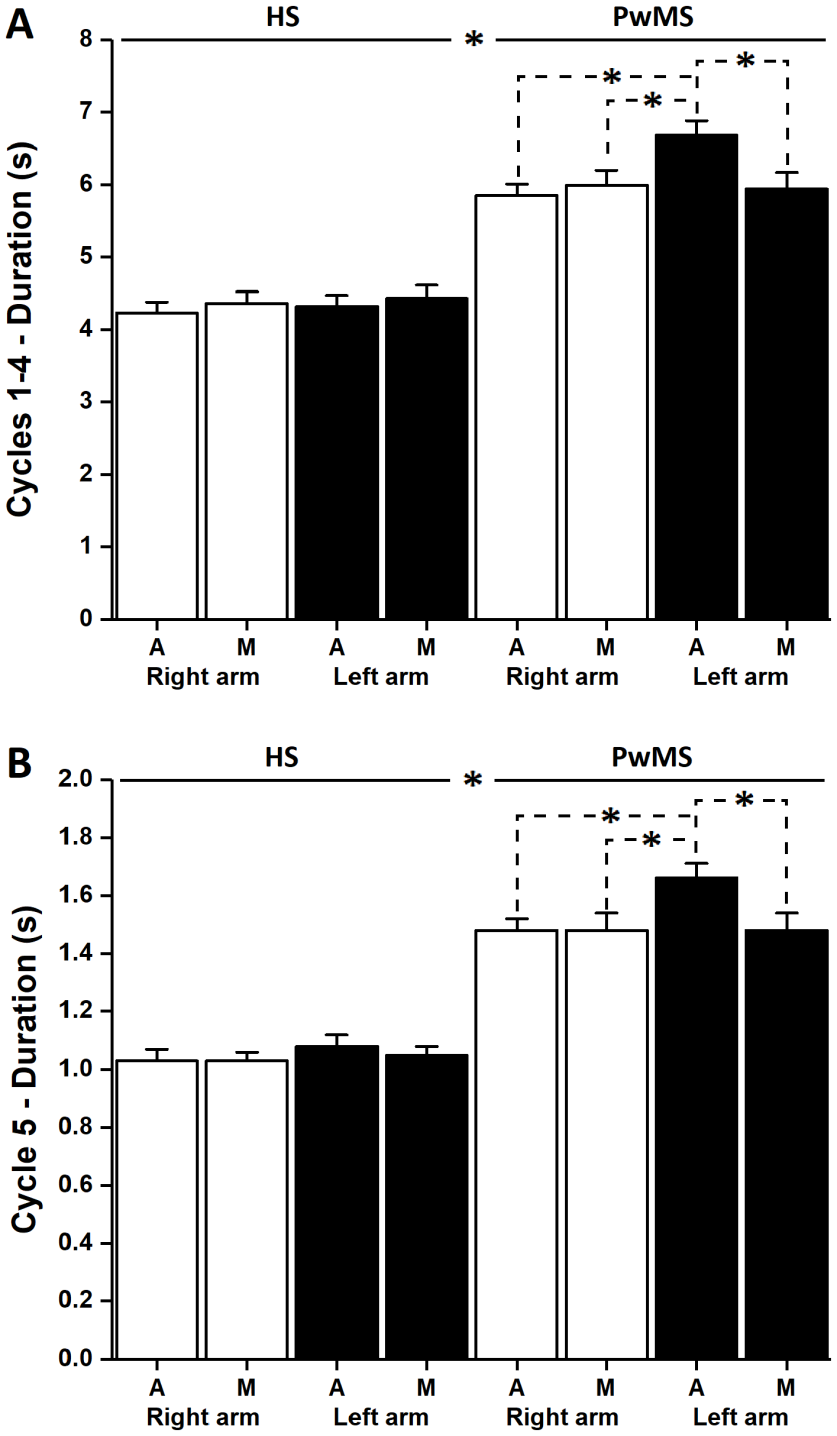
Duration of the actual and mental trials. **(A)** Shows the average values and standard deviation of the duration of the first four actual and mental movements. Significant differences between the HS and PwMS, between the right and left arms, and between actual and mental trials can be observed for the patient group (stars; *p* < 0.01). **(B)** Shows the average values and standard deviation of the duration of the fifth actual movement of the actual and mental trials. Significant differences can be observed between HS and PwMS, between the right and left arms, and between actual and mental trials (stars; *p* < 0.01) for the patient group.

Finally, a significant interaction between group, type, and arm was found [F (1,136) = 6.17, *p* < 0.05]. *Post hoc* analysis revealed a significant difference between groups for all conditions considered (always *p* < 0.001); furthermore, significant differences were found for PwMS between actual and mental durations with the left arm (*p* < 0.001) and between actual durations with the left arm and both actual and mental durations with the right arm (*p* < 0.01) (PwMS actual right: 5.85 ± 0.16 s, actual left: 6.68 ± 0.20 s, mental right: 5.99 ± 0.21 s, mental left: 5.94 ± 0.23 s; HS actual right: 4.23 ± 0.15 s, actual left: 4.32 ± 0.15 s, mental right: 4.36 ± 0.16 s, mental left: 4.43 ± 0.18 s) (Figure 2A).

**Figure 3:**
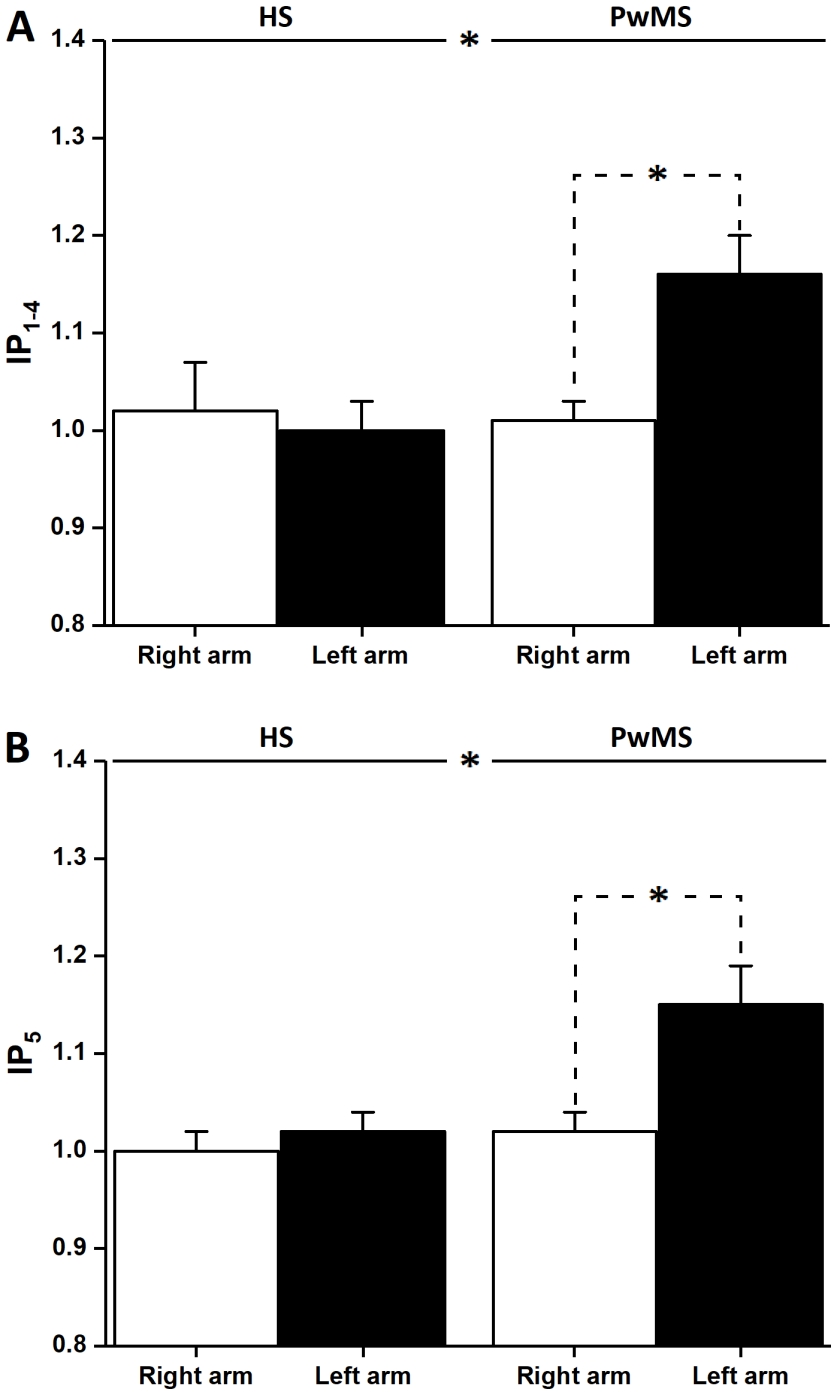
Anisochrony and MI priming effect in PwMS. **(A)** Shows the average values and standard deviation of the ratio of the duration of the first four actual movements and the four mental movements. Significant differences can be observed between HS and PwMS and between the right and left arms for the patient group (stars; *p* < 0.05); although not shown, significant differences were also present between the left arm of PwMS and the right and left arms of HS (for both *p* < 0.01). **(B)** Shows the average values and standard deviation of the ratio of the duration of the fifth actual movement of actual and mental trials. Significant differences can be observed between HS and PwMS (stars; *p* < 0.01) and between the right and left arms (stars; *p* < 0.0001) for the patient group; although not shown, significant differences were also present between the left arm of PwMS and the right and left arms of HS (for both, *p* < 0.0001).

The statistical analysis of IP_1–4_ provided valuable information about MI ability (Figure 3A). ANOVA revealed a main effect of group [PwMS: 1.09 ± 0.02; HS: 1.00 ± 0.02; *F* (1,136) = 4.71, *p* < 0.05] and a significant interaction effect between group and arm [F (1,136) = 5.11, *p* < 0.05]. *Post hoc* analysis showed that IP_1–4_ was significantly higher in the left arm of PwMS (1.16 ± 0.04) than in the right arm of PwMS (1.01 ± 0.02) (*p* < 0.05) and in the right and left arms of HS (1.02 ± 0.05 and 1.00 ± 0.03, respectively, for both *p* < 0.01).

### Effect of mental simulation on actual execution

A randomization of all conditions (i.e., arm, type, and target size) was introduced to control for the potential effect of the sequence disposition of actual and mental trials on the performances shown in the fifth actual cycle.

On the fifth cycle of both actual and mental trials, ANOVA revealed main effects of group [PwMS: 1.53 ± 0.03 s; HS: 1.05 ± 0.02 s; *F* (1,136) = 147.90, *p* < 0.001], target size [small: 1.32 ± 0.03 s; large: 1.22 ± 0.03 s; F (1,136) = 5.79, *p* < 0.05], and type [actual: 1.30 ± 0.03 s; mental: 1.25 ± 0.03 s; F (1,136) = 10.30, *p* < 0.01].

Moreover, we found a significant interaction between group and type [F (1,136) = 6.85, *p* < 0.01]. *Post hoc* analysis revealed that the fifth cycle in both actual and mental trials was significantly longer in PwMS than in HS (PwMS actual: 1.57 ± 0.04 s, mental: 1.48 ± 0.04 s; HS actual: 1.05 ± 0.03 s, mental: 1.04 ± 0.02 s; *p* < 0.001); only in PwMS, the fifth cycle in actual trials was significantly longer than the fifth cycle in mental trials (*p* < 0.001). A significant interaction between arm and type was present [F (1,136) = 9.13, *p* < 0.01]. *Post hoc* analysis revealed that the fifth cycle was significantly longer in actual trials performed with the left arm than in mental trials with both right (*p* < 0.05) and left (*p* < 0.001) arms (right actual: 1.24 ± 0.04 s, mental: 1.24 ± 0.04 s; left actual: 1.35 ± 0.05 s, mental: 1.23 ± 0.04 s).

There was a significant interaction between group, type, and arm [F (1,136) = 5.45, *p* < 0.05]. *Post hoc* analysis revealed a significant difference between groups for all conditions considered (always *p* < 0.001); furthermore, significant differences were found only for PwMS between actual and mental durations with the left arm (*p* < 0.001) and between actual durations with the left arm and both actual and mental durations with the right arm (*p* < 0.05), suggesting a priming effect of MI on actual execution in the non-dominant arm (PwMS actual right: 1.48 ± 0.04 s, actual left: 1.66 ± 0.05 s, mental right: 1.48 ± 0.06 s, mental left: 1.48 ± 0.06 s; HS actual right: 1.03 ± 0.04 s, actual left: 1.08 ± 0.04 s, mental right: 1.03 ± 0.03 s, mental left: 1.05 ± 0.03 s) (Figure 2B).

Statistical analysis of IP_5_ confirmed these results (Figure 3B). ANOVA revealed a main effect of group [PwMS: 1.08 ± 0.02; HS: 1.01 ± 0.02; F (1,136) = 8.71, *p* < 0.01] and arm [right: 1.01 ± 0.02; left: 1.08 ± 0.02; F (1,136) = 7.79, *p* < 0.01] and a significant interaction between group and arm [F (1,136) = 5.35, *p* < 0.05]. *Post hoc* analysis showed that IP_5_ was significantly higher in the left arm in PwMS (1.15 ± 0.04) than in the right arm in PwMS (1.02 ± 0.02) (*p* < 0.001) and in both the right and left arms in HS (1.00 ± 0.02 and 1.02 ± 0.02, respectively, for both *p* < 0.001).

## Discussion

To the best of our knowledge, this is the first study to investigate the potential role of MI as a motor priming method in MS. We employed a modified version of the task used in Tacchino et al. (2013); here, an actual movement follows the first four movements in both actual and mental tasks.

As expected, the analysis of the temporal characteristics of the first four movements in actual and mental trials confirmed previous results (Tacchino et al., 2013). Indeed, we found that PwMS executed both actual and mental arm movements significantly slower than HS, as a consequence of the general motor and cognitive slowing due to MS and task complexity, both of which influence action representation and actual execution (Guillot and Collet, 2005; Bonzano et al., 2013; Giovannoni et al., 2016; Di Giovanni et al., 2021).

Moreover, consistent with the literature, we confirmed the presence of isochrony in HS (Decety et al., 1989; Skoura et al., 2008) and anisochrony in PwMS (Tacchino et al., 2013, 2018; Podda et al., 2020). As shown by the statistical analysis of the duration of the first 4 cycles, the results confirm previous findings showing that anisochrony in PwMS is mostly due to actual execution with the non-dominant arm being significantly slower than actual execution with the dominant arm and mental simulation with both arms (Tacchino et al., 2013). Indeed, even the actual performance of a simple task with the non-dominant arm would be challenging for PwMS due to decreased coordination and accuracy compared to the dominant arm (Lamers et al., 2013). However, this increased difficulty would have no impact on mental execution with the non-dominant arm; PwMS would be able to mentally represent movement and action context as with the dominant arm, resulting in a temporal uncoupling in the non-dominant arm (Guillot and Collet, 2005).

Most importantly and novelly, four mental movements with the non-dominant arm were able to speed up a subsequent actual movement when compared with the actual trials. Thus, it could be argued that MI has a priming effect on actual execution.

### Interpretation of the MI priming effect

The acceleration of the fifth movement after mental simulation with the non-dominant (i.e., left) arm could be interpreted as a priming effect exerted by MI on the actual execution. The presence and functioning of the inverse and forward internal models would explain this result (Wolpert and Kawato, 1998; Hesslow, 2002).

**Figure 4:**
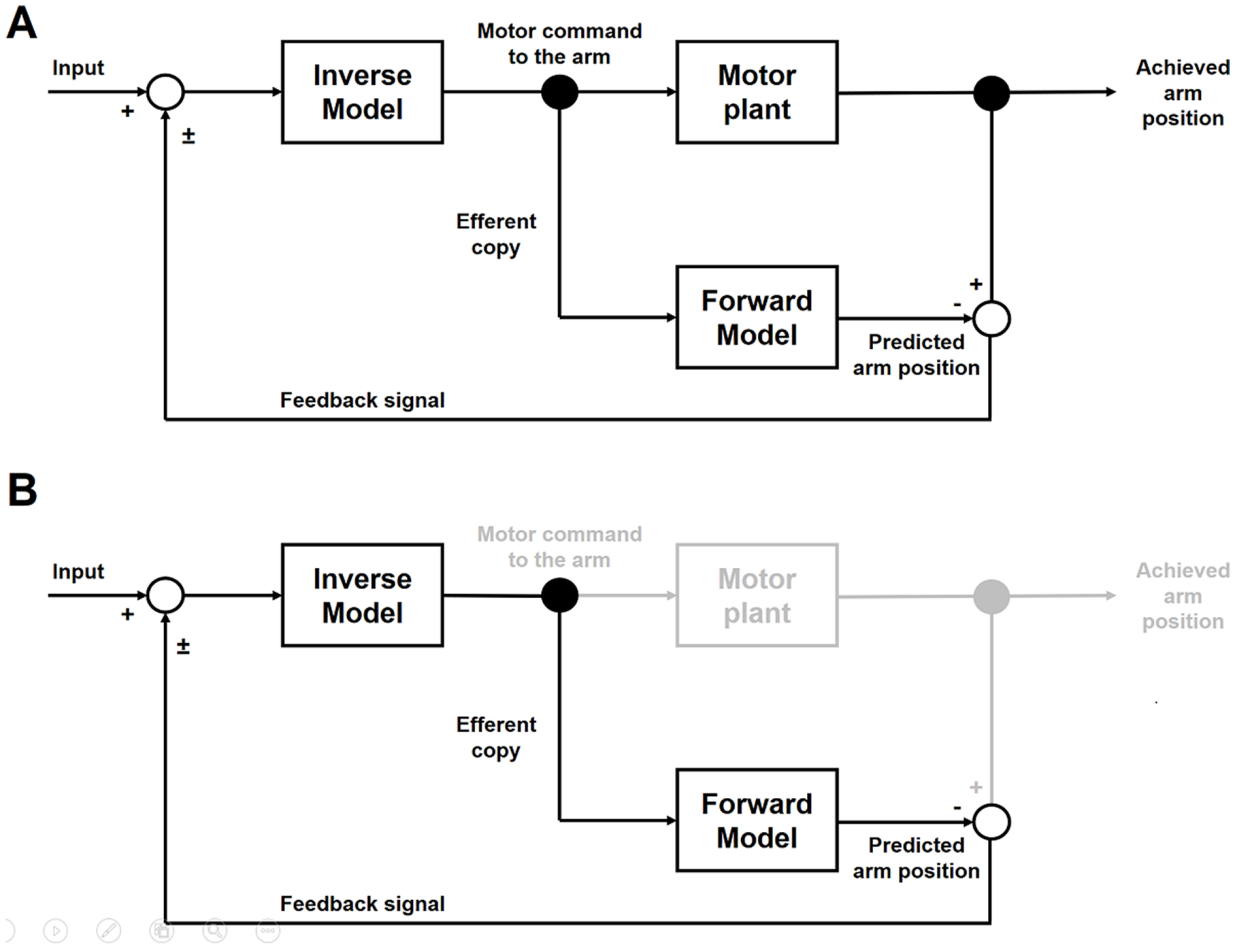
Inverse and Forward internal models. **(A)** Shows the inverse and forward models at work during actual movements. **(B)** Shows the inverse and forward models at work during mental movements.

Briefly (Figure 4), the inverse internal model uses sensory information - somatosensory input regarding the arm position and visuospatial input regarding the action context - in order to estimate the motor command needed to control the arm and bring it into the position required by the task (i.e., motor plant). Contextually, an efferent copy of the motor command is sent to the forward internal model for predicting the arm position.

During actual movements, the output of the forward model - the predicted arm position (i.e., the sensory consequences of the movement) - is compared with the achieved arm position. Differences between them provide feedback to improve the movement for the next iteration of the internal models (Figure 4A).

During mental movements, although the inverse model correctly prepares the motor command, these are blocked and the motor plant is not activated; nevertheless, the efferent copy is available and, consequently, the forward model is still able to predict the arm position (Figure 4B).

In HS, the correct functioning of the inverse and forward internal models and the motor plant would guarantee similar temporal estimations between actual and mental movements with both arms.

As expected, MS would slow down the functioning of the internal models and the motor plant, and, consequently, longer durations of actual and mental movements would be similarly observable in both arms. However, lower motor coordination and accuracy when the task is performed with the non-dominant arm would be responsible for the even longer durations observed during the actual movements. In addition, the comparison between the achieved and predicted arm positions would generate an increased error and incorrect feedback that would weaken the inverse model’s functioning and further would slow down the actual movement execution.

On the contrary, during MI with the non-dominant arm, the motor plant is not activated, and mental movements are generated with durations similar to those observed during actual and mental movements with the dominant arm. Thus, after the four mental cycles, the inverse model has more reliable inputs available, and the motor commands generated would allow actual movements to be performed as long as they can be observed with the dominant arm.

The priming effect could be due to the more efficient functioning of both internal models during mental movements with the non-dominant arm. This finding suggests that a few mental repetitions of an action are sufficient to exert a priming effect on the actual execution of the same action. However, we could interpret this phenomenon in two different ways.

On the one hand, MI would prevent the corruption of the functioning of the internal models and, consequently, would activate a more reliable action representation; in our case, it would allow the execution of the fifth actual movement after the four mental movements as long as it could be observed with the dominant arm.

On the other hand, we could hypothesize that the MI priming effect also emerges following a motor learning process attributed to the repetition of the covert stimulation; it would emerge only with the non-dominant arm, whereas in the case of the dominant arm, more mental repetitions would be necessary to observe changes in the action representation capable of producing an effect on the actual execution (Hauptmann and Karni, 2002; Louis et al., 2008). After all, numerous reports have suggested that MI practice is an effective means of producing functional and stable changes within the motor action system by inducing motor skill learning and improving actual execution performance (Jackson et al., 2001; Jeannerod, 2001). MI practice has been shown to be more effective than no practice and less effective than physical practice (Driskell et al., 1994), whereas the combination of mental and physical practice has been suggested to be as effective as or superior to physical practice (Jackson et al., 2001; Dickstein and Deutsch, 2007; Simmons et al., 2008; Malouin et al., 2013; Frank and Schack, 2017; Hanson and Concialdi, 2019; Seebacher et al., 2019; Kahraman et al., 2020).

### Study limitations and future perspectives

The main limitation of the study is the lack of knowledge about brain activations while the subjects were performing the task. For example, the use of MRI and fMRI could give information on brain areas that may be active during MI and actual movements in both actual and mental trials. Indeed, data from fMRI could highlight significant differences in brain activations during actual movements after MI in PwMS. Thus, further studies using tasks of higher complexity and advanced neurophysiological techniques (e.g., MRI, fMRI, and transcranial magnetic stimulation) could better clarify the behavioral and neural correlates of the MI-based priming effect in PwMS; indeed, probing the mechanisms underpinning this phenomenon could make known how MI primes action execution in MS.

These investigations could be crucial for the introduction of MI into MS clinical practice. Indeed, MI is a promising potential rehabilitation method for PwMS because rehabilitative training using MI is relatively easy and effective in improving motor performance, as recently shown in other clinical populations (primarily in stroke and Parkinson’s disease). However, for this purpose, it is imperative to conduct robust research (i.e., randomized controlled trials), for example, to investigate the effect of MI as an add-on to a traditional physical intervention. In addition, objective control measures specific to MS should be used to quantify the relevance of the changes induced by MI training and the eventual positive impact on the trained function. Furthermore, in this context, MRI could be used to evaluate the differences between neural networks stimulated in PwMS before and after MI training.

## Conclusion

MI has already gained attention as a promising additional rehabilitation method for neurological disorders such as stroke, Parkinson’s disease, spinal cord injury, and amputation. Our findings shed new light on the role of MI in MS and suggest that the potential use of MI as a new motor-cognitive tool for the neurorehabilitation of PwMS should be investigated.

## Data availability statement

The raw data supporting the conclusions of this article will be made available by the authors, without undue reservation.

## Ethics statement

The studies involving humans were approved by the Comitato Etico Regionale, Ospedale San Martino. The studies were conducted in accordance with the local legislation and institutional requirements. The participants provided their written informed consent to participate in this study.

## Author contributions

AT: substantial contributions to the conception and design of the work, acquisition, analysis, interpretation of data for the work, and drafting the work. LP, JP, MM, and AB: acquisition, analysis, interpretation of data for the work, and critical revision of the work for important intellectual content. MAB: substantial contributions to the conception of the work. MB and GB: substantial contributions to the conception and design of the work, critical revision of the work for important intellectual content. All authors have given approval for publication of the content and have agreed to take responsibility for all aspects of the work, ensuring that questions relating to the accuracy or integrity of any part of the work were appropriately investigated and resolved.

## Conflict of interest

The authors declare that the research was conducted in the absence of any commercial or financial relationships that could be construed as a potential conflict of interest.

## Publisher’s note

All claims expressed in this article are solely those of the authors and do not necessarily represent those of their affiliated organizations, or those of the publisher, the editors and the reviewers. Any product that may be evaluated in this article, or claim that may be made by its manufacturer, is not guaranteed or endorsed by the publisher.
